# Brain Mechanisms of Exercise-Induced Hypoalgesia: To Find a Way Out from “Fear-Avoidance Belief”

**DOI:** 10.3390/ijms23052886

**Published:** 2022-03-07

**Authors:** Katsuya Kami, Fumihiro Tajima, Emiko Senba

**Affiliations:** 1 Department of Rehabilitation, Faculty of Wakayama Health Care Sciences, Takarazuka University of Medical and Health Care, Wakayama 640-8392, Japan; 2 Department of Rehabilitation Medicine, Wakayama Medical University, Wakayama 641-8509, Japan; tajibun@gmail.com (F.T.); emikosenba@gmail.com (E.S.); 3 Department of Physical Therapy, Osaka Yukioka College of Health Science, Osaka 567-0801, Japan

**Keywords:** sciatic nerve ligation, exercise-induced hypoalgesia, mesocorticolimbic system, brain reward system, fear-avoidance model, voluntary running, chronic pain

## Abstract

It is well known that exercise produces analgesic effects (exercise-induced hypoalgesia (EIH)) in animal models and chronic pain patients, but the brain mechanisms underlying these EIH effects, especially concerning the emotional aspects of pain, are not yet fully understood. In this review, we describe drastic changes in the mesocorticolimbic system of the brain which permit the induction of EIH effects. The amygdala (Amyg) is a critical node for the regulation of emotions, such as fear and anxiety, which are closely associated with chronic pain. In our recent studies using neuropathic pain (NPP) model mice, we extensively examined the association between the Amyg and EIH effects. We found that voluntary exercise (VE) activated glutamate (Glu) neurons in the medial basal Amyg projecting to the nucleus accumbens (NAc) lateral shell, while it almost completely suppressed NPP-induced activation of GABA neurons in the central nucleus of the Amyg (CeA). Furthermore, VE significantly inhibited activation of pyramidal neurons in the ventral hippocampus-CA1 region, which play important roles in contextual fear conditioning and the retrieval of fear memory. This review describes novel information concerning the brain mechanisms underlying EIH effects as a result of overcoming the fear-avoidance belief of chronic pain.

## 1. Introduction

**Figure 1:**
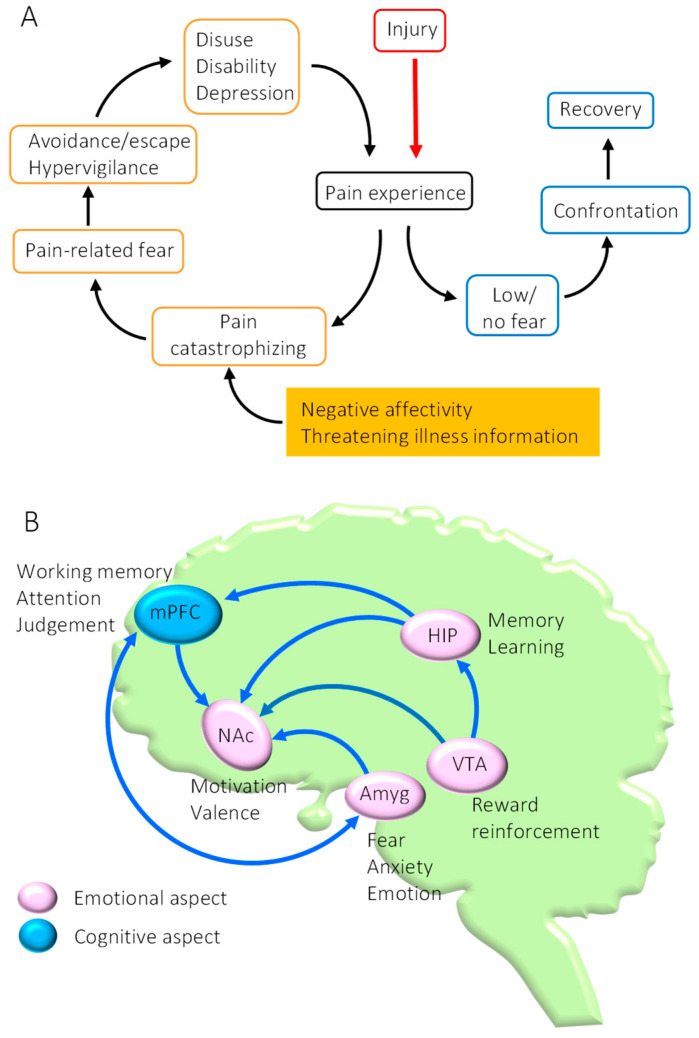
Fear-avoidance model and the mesocorticolimbic system. (**A**) Fear-avoidance model. (**B**) The mesocorticolimbic system, consisting of the mPFC, the Amyg, the NAc, and the VTA, constitutes a group of brain areas involved in the cognitive and emotional aspects of pain. Dysfunction of these regions plays a critical role in the chronification of pain. mPFC: medial prefrontal cortex; Amyg: amygdala; NAc: nucleus accumbens; HIP: hippocampus; VTA: ventral tegmental area.

The biopsychosocial model of pain chronification emphasizes the importance of psychosocial factors in addition to biological factors in the development and maintenance of pain [1]. Among the psychological factors related to pain, anxiety and fear (emotional aspects of pain) in particular may play important roles in establishing chronic pain [2]. In addition, pain catastrophizing is common in chronic pain patients, where anxiety and fear of pain are further reinforced by negative affectivity and threatening illness information [3]. Consequently, when the attention of patients is restricted solely to their pain, they avoid any behaviors that predict induction of pain, leading to a dramatic reduction in physical activity (establishment of inactivity). Physical inactivity can result in several disuse syndromes, such as degradation of skeletal muscle function, depression, and social withdrawal, and can produce a vicious cycle that is reinforced by persistent pain [4,5]. The fear-avoidance model, advocated by Lethem et al., suggests that chronic pain is established through a vicious cycle consisting of anxiety, fear, and avoidance behaviors related to pain (Figure 1A) [6]. Leeuw et al. have also reported that fear-induced behaviors aimed at avoiding pain can become risk factors that lead to worsening of chronic lower back pain [7]. These findings suggest that effective therapies to alleviate chronic pain should include a well-defined method that reduces avoidance behaviors caused by pain-related fear and anxiety. A possible therapy fulfilling these terms is exercise therapy. However, the brain mechanisms that underlie exercise-induced analgesic effects (exercise-induced hypoalgesia (EIH)), especially concerning the emotional aspect of pain, are still not sufficiently understood. In this review, we will discuss how plastic changes in the mesocorticolimbic system could affect chronic pain. In addition, we summarize our recent studies which have demonstrated that voluntary exercise (VE) can not only normalize the mesocorticolimbic system that has fallen into dysfunction in the chronic pain state but can also facilitate the extinction of fear memories acquired through contextual fear conditioning. These findings provide novel insights into the brain mechanisms producing EIH effects and may enable chronic pain patients to overcome fear-avoidance behaviors and halt the vicious cycle of pain chronification.

## 2. Dysfunction of the Mesocorticolimbic System in Chronic Pain State

**Figure 2:**
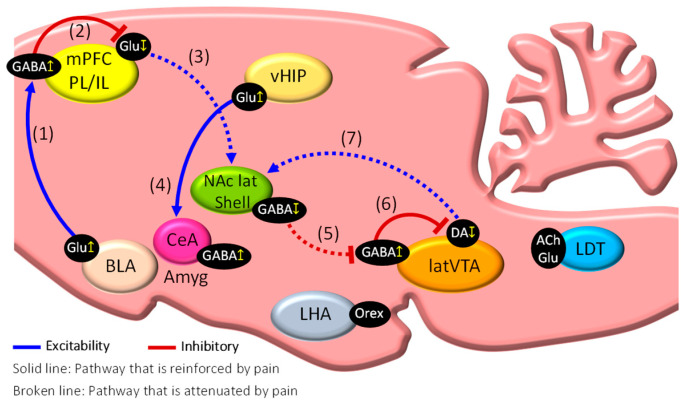
Dysfunction of the mesocorticolimbic system induces pain chronification. Schematic diagram showing the possible mechanisms that induce pain chronification. Our recent studies using NPP model mice have indicated that dysfunction of the mesocorticolimbic system promotes pain chronification. Hyperexcitation of Glu neurons in the BLA by noxious stimulation can inhibit mPFC Glu neurons (2) through activated mPFC GABA interneurons (BLA–mPFC pathway) (1), resulting in the inhibition of NAc GABA neurons (mPFC–NAc pathway) (3). This hyperexcitation by noxious stimulation can also excite Glu neurons in the ventral HIP (vHIP) that subsequently activate GABA neurons in the CeA (vHIP–CeA pathway) (4). During a painful episode, inactivation of the GABA-positive medium spiny neurons (MSNs) in the NAc lat shell promote the disinhibition of GABA neurons in the latVTA (5). Consequently, activated GABA neurons in the latVTA then inhibit the VTA DA neurons projecting to the NAc lat shell (6). As a result, the release of DA into the NAc lat shell (latVTA–NAc pathway) is downregulated (7), causing an increase in pain (see text for further details). mPFC: medial prefrontal cortex; BLA: basolateral amygdala; CeA: central nuclei of the amygdala; Amyg: amygdala; NAc lat shell: nucleus accumbens lateral shell; vHIP: ventral hippocampus; latVTA: lateral ventral tegmental area; LDT: laterodorsal tegmental nucleus; LHA: lateral hypothalamus; GABA: GABAergic neurons; Glu: glutamatergic neurons; DA: dopaminergic neurons; Orex: orexinergic neurons; ACh: cholinergic neurons.

The research findings of human brain imaging studies have indicated that chronification of pain is produced via plastic changes in brain regions closely associated with the emotional aspects of pain, such as anxiety and fear. These findings suggest that the intensity of noxious stimulation is not the only cause of chronic pain. In particular, the mesocorticolimbic system of the brain, consisting of the medial prefrontal cortex (mPFC), amygdala (Amyg), nucleus accumbens (NAc), hippocampus (HIP), and ventral tegmental area (VTA), plays an essential role in the chronification of pain because these regions are closely associated with cognitive and emotional aspects of pain [8,9,10] (Figure 1B). Our findings have demonstrated that dysfunction of the mesocorticolimbic system may play a critical role in the chronification of pain [11]. These contributory factors and pathways are summarized in Figure 2.

The mammalian mPFC is divided into the prelimbic cortex (PL) and the infralimbic cortex (IL). The mPFC is strongly innervated by the Amyg and HIP and acts as a node for cognitive functions, such as working memories and decision-making, in addition to its role as a top-down controller of pain through robust projections to the NAc, Amyg, and periaqueductal gray (PAG). Pain-induced activation of glutamatergic (Glu) neurons in the basolateral amygdala (BLA) can activate parvalbumin (PV)-expressing GABA-positive interneurons in the mPFC (Figure 2(1)), whereas pharmacological inhibition of the overactivated BLA results in the disinhibition of the Glu neurons in the PL, thereby inhibiting pain-related behaviors [12]. Accordingly, optogenetic inhibition of PV-expressing GABA interneurons has been revealed to reduce pain-related behaviors in rodent models of neuropathic pain (NPP), while optogenetic activation aggravated pain-related behaviors [13]. These findings indicate that feedforward inhibition of mPFC Glu neurons via activation of inhibitory GABA interneurons in the mPFC is a potent mechanism of chronification and aggravation of pain (Figure 2(2)). A recent study using neuropathic pain model mice revealed excitation of pyramidal neurons in the second/third layers and decreased excitation of pyramidal neurons in the fifth layer of the PL, while the equivalent neurons in the IL were unaffected [14]. In addition, optogenetic activation of Glu neurons in the fifth layer of the PL was demonstrated to inhibit several behaviors related to pain and depression [15].

The Amyg is mainly composed of the BLA, the intercalated cell mass (ITC), and the central nucleus of the amygdala (CeA). The CeA is further subdivided into the CeL (lateral division), the CeM (medial division), and the CeC (capsular division). Noxious information that was modified by past painful experiences and mood states via the central nervous system is received into the CeA via the BLA (BLA–CeA pathway). On the other hand, noxious information derived from peripheral tissues and organs is directly received into the lateral parabrachial nucleus (lPBN) via the spinal dorsal horn, from where it is sent onwards to the CeC of the CeA (lPBN–CeA pathway). It has been demonstrated that Amyg neurons are overactivated in experimental animals suffering from various pain states, including visceral pain, formalin-induced inflammatory pain, acid-induced muscle pain, arthritic pain, and neuropathic pain [16]. Hyperexcitation of Glu neurons in the BLA by noxious stimulation can inhibit mPFC Glu neurons (Figure 2(2)) through activated mPFC GABA interneurons (BLA–mPFC pathway) (Figure 2(1)) (feedforward inhibition), resulting in the inhibition of NAc GABA neurons (mPFC–NAc pathway) (Figure 2(3)). This hyperexcitation by noxious stimulation can also excite Glu neurons in the ventral HIP (vHIP) that subsequently activate GABA neurons in the CeA (vHIP–CeA pathway) (Figure 2(4)) [17]. All these events may be critical to establish chronic pain conditions. On the other hand, activation of the lPBN–CeA pathway produces aversive behaviors, such as anxiety and depression, while activation of the BLA–CeA pathway induces reward-related behaviors, reduces anxiety and depression, and promotes the inhibition of pain [18]. Therefore, the different neuronal types of the BLA, each with different projection targets, act to differentially control aversive and reward-related behaviors closely associated with pain and hypoalgesia, respectively [19].

The HIP (hippocampus) is divided into the dorsal and ventral HIP (vHIP), which play important roles in several cognitive and emotional functions, including spatial memory, learning, fear, and anxiety. The vHIP is strongly innervated by the mPFC and the BLA and sends projections to the mPFC, the BLA, and the CeA that contribute to the expression of fear memories and anxiety [17]. Neurogenesis in the hippocampal dentate gyrus (DG) is known to be inhibited in mouse models of NPP [20], a finding that may explain the decrease in HIP volume observed in chronic pain patients [20]. Levels of tumor necrosis factor-α (TNF-α) are increased in the HIP of NPP model mice. It has been reported that administration of TNF-α into the HIP of wild-type rats promotes pain-related behaviors similar to those seen in NPP model rats, while reduced expression of TNF receptor-1 in NPP model mice is associated with a decrease in pain-related behaviors [21]. A preliminary study by our group detected a significant increase in activated Glu neurons in the vHIP–CA1 regions of NPP model mice (data not shown). A study indicated that the ability to extinguish contextual fear memories is significantly weakened in NPP model mice [20]. These findings suggest that NPP-induced activation of Glu neurons in the vHIP–CA1 regions may play a critical role in promoting “fear-avoidance behaviors”.

The brain’s reward system consists of a projection pathway of dopamine (DA) neurons from the lateral VTA (latVTA) to the NAc lateral shell (NAc lat shell), and activation of this pathway can promote positive behaviors and reinforce learning and desire. The NAc is a core brain region of the reward system that controls valence (an emotional value to motivate us to select appropriate behaviors) and allows us to adapt to our external environment. Production of DA in the latVTA is inhibited in chronic pain patients, and this inhibition can contribute to the development of depression [22]. In addition, it has been shown that DA levels in the latVTA are significantly decreased in the rat NPP model, resulting in mechanical hyperalgesia. Furthermore, injection of apomorphine (a D2/D1 agonist) directly into the NAc of NPP rats can prevent this [23,24]. Alternatively, VTA DA neurons receive robust inputs from GABA interneurons located in the latVTA. During a painful episode, inactivation of the GABA-positive medium spiny neurons (MSNs) in the NAc lat shell promote the disinhibition of GABA neurons in the latVTA (Figure 2(5)). Consequently, activated GABA neurons in the latVTA then inhibit the VTA DA neurons projecting to the NAc lat shell (Figure 2(6)) [25]. As a result, the release of DA into the NAc lat shell (latVTA–NAc pathway) is downregulated (Figure 2(7)), causing an increase in pain. Therefore, inhibition of DA neurons in the latVTA via GABA neurons may be a key mechanism in pain chronification in chronic pain patients.

## 3. Brain Mechanisms of Exercise-Induced Hypoalgesia (EIH)

Meta-analyses and experimental animal studies over the past 30 years have demonstrated that exercise, such as running or swimming, can promote analgesic effects (EIH) and that the ability of exercise to improve chronic pain is well established in humans and animals [26,27,28]. It has been proposed that EIH may be the result of several plastic changes in the peripheral nerves, spinal cord, and brain stem following different types of exercise [29,30]. These observations suggest that exercise is a valid intervention that is recommended prior to pharmacological therapy to improve chronic pain, because compared with pharmacological therapy, exercise therapy can be performed without any side effects. Moreover, opportunities to augment the levels of individual physical activity are commonly presented in daily life, such as commuting, cleaning, shopping, and household chores. Thus, the ease and convenience of exercise therapy gives it a great advantage over other interventions.

When inflammatory pain model mice were kept in an enriched environment with a running wheel, significant improvements in perceptual, emotional, and cognitive brain functions were observed in active mice compared to sedentary mice [31]. It has been shown that voluntary exercise (VE) induces anti-depressant effects through an increase in DA levels in the mPFC, and that VE can also enhance performance in cognitive tasks [32]. Furthermore, Pitcher et al. reported that VE can reduce both pain- and stress-related behaviors [33]. We observed that VE, using a voluntary wheel running apparatus, in NPP model mice could enhance EIH effects compared to those subjected to forced exercise, such as treadmill running [34]. These beneficial effects of exercise may be mediated by the activation of the brain’s reward system and normalization of the mesocorticolimbic system.

The role of endogenous opioids in producing EIH effects has been reported by Stagg et al. [35]. They indicated that in NPP model animals, exercise increased the content of b-endorphin and met-enkephalin in the midbrain periaqueductal gray (PAG) and rostral ventromedial medulla (RVM), which are the two principal areas of the brainstem responsible for the descending modulation of pain. In addition, Bobinski et al. [36] showed that low-intensity treadmill running in sciatic nerve-crushed mice increases serotonin (5-HT) concentration and expression of its receptors (5HT-1B, 2A, 2C) and decreases the expression of the serotonin transporter in the brainstem, with a corresponding reduction in mechanical hyperalgesia. Furthermore, treadmill running in NPP model rats restored the expression of 5HT receptors in spinal cord dorsal horns [37]. In addition, the role of myokines secreted by skeletal muscle, including brain-derived neurotrophic factor (BNDF), irisin, cathepsin B, and insulin-like growth factor-I (IFG-I), in inducing EIH effects was extensively reviewed by Wang et al. [38]. Thus, well-characterized analgesic mechanisms of EIH effects have been explained by the endogenous opioid system, serotonergic system, and myokine system. Both the endogenous opioid/serotonergic system and mesocorticolimbic system may play important roles to induce EIH effects, having implications with each other [8,39,40].

**Figure 3:**
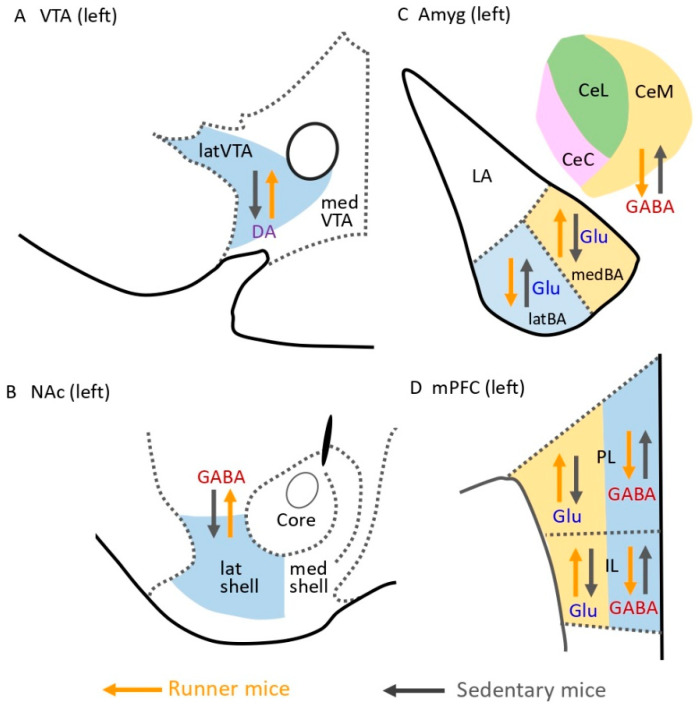
Effects of voluntary exercise and sedentary behavior on neuronal activity in the mesocorticolimbic system of NPP model mice. Schematic diagrams showing changes to (**A**) DA neurons in the latVTA, (**B**) GABA neurons in the NAc lat shell, (**C**) Glu and GABA neurons in the Amyg, and (**D**) Glu and GABA neurons in the mPFC in runner and sedentary mice. Voluntary running mice (runner mice) received partial sciatic nerve ligation (PSL) surgery and were then reared in cages equipped with a running wheel for 2 weeks. On the other hand, sedentary mice were kept in the cages with an immobilized running wheel for 2 weeks after PSL surgery. The left side of the areas is shown because PSL surgeries were performed on the right side. VTA: ventral tegmental area; latVTA: lateral ventral tegmental area; medVTA: medial ventral tegmental area; Amyg: amygdala; LA: lateral amygdala; latBA: lateral basal amygdala; medBA: medial basal amygdala; CeM: medial division of CeA; CeC: capsular division of CeA; CeL: lateral division of CeA; NAc: nucleus accumbens; lat shell: nucleus accumbens lateral shell; med shell: nucleus accumbens medial shell; mPFC: medial prefrontal cortex; PL: prelimbic cortex; IL: infralimbic cortex.

**Figure 4:**
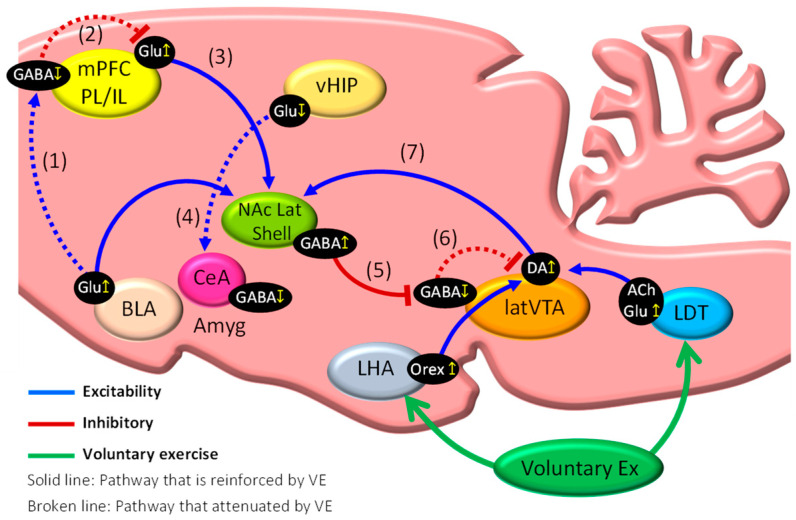
Effects of voluntary exercise on mesocorticolimbic system. Schematic diagram showing brain mechanisms implicated in EIH effects. Our recent studies using NPP model mice indicated that functional normalization of the mesocorticolimbic system following voluntary running can alleviate chronic pain. VE in NPP model mice inhibits GABA interneurons in the PL and IL of the mPFC, probably via inhibition of the BLA–mPFC pathway (1), which disinhibits Glu neurons working in the mPFC–NAc pathway (2), thereby activating NAc GABA neurons (3). Significant inhibition of GABA neurons in the CeA following VE may be promoted via inhibition of Glu neurons working in the vHIP–CeA pathway (4). Activation of GABA neurons in the NAc lat shell via activation of the mPFC–NAc, BLA–NAc, and latVTA–NAc pathways may inhibit GABA interneurons located in the latVTA (5), which results in disinhibition of VTA DA neurons (6) and, therefore, upregulation of DA release into the NAc lat shell (7) (see text for further details). These findings provide a rationale for exercise therapy in chronic pain patients. mPFC: medial prefrontal cortex; BLA: basolateral amygdala; CeA: central nuclei of amygdala; Amyg: amygdala; NAc lat shell: nucleus accumbens lateral shell; vHIP: ventral hippocampus; latVTA: lateral ventral tegmental area; LDT: laterodorsal tegmental nucleus; LHA: lateral hypothalamus; GABA: GABAergic neurons; Glu: glutamatergic neurons; DA: dopaminergic neurons; Orex: orexinergic neurons; ACh: cholinergic neurons.

Wakaizumi et al. [41] examined the importance of the mesocorticolimbic system in the induction of EIH using the Designer Receptors Exclusively Activated by Designer Drugs (DREADD) system, in which they suggested that the dopaminergic pathway from the VTA to the NAc is critically involved in anti-nociception in NPP model mice under low-intensity exercise. It is well known that VE can activate the brain reward system [42], which suggests that activation of the mesocorticolimbic system including the brain reward system may play a critical role to produce EIH effects. In addition, our studies have shown that the mesocorticolimbic system plays an important role in pain in NPP mice, and we will now propose several brain mechanisms that underlie EIH effects [11,34,43]. The main findings of these studies are summarized in Figure 3, while the mechanisms suggested to control EIH effects are indicated in Figure 4. VE in NPP model mice activates Glu neurons in the medial basal nucleus of the Amyg (medBA), which projects to the NAc lat shell (medBA–NAc pathway) (Figure 4) [43]. Moreover, VE in NPP model mice inhibits GABA interneurons in the PL and IL of the mPFC, probably via inhibition of the BLA–mPFC pathway (Figure 4(1)), which disinhibits Glu neurons working in the mPFC–NAc pathway (Figure 4(2)), thereby activating NAc GABA neurons (Figure 4(3)). On the other hand, significant inhibition of GABA neurons in the CeA following VE may be promoted via inhibition of Glu neurons working in the vHIP–CeA pathway (Figure 4(4)), which may act to reduce discomfort and fear associated with chronic pain [43].

Our previous studies [11,34] and preliminary observation (Figure 3B) indicated that VE in NPP model mice induces significant activation of tyrosine hydroxylase-positive DA neurons in the latVTA and GAD67-positive GABA neurons in the NAc lat shell, respectively. Furthermore, VE in NPP model mice induces both activation of cholinergic and non-cholinergic neurons in the laterodorsal tegmental nucleus (LDT) (LDT–latVTA pathway) and activation of orexin neurons in the lateral hypothalamus (LHA) (LHA–latVTA pathway), indicating that both pathways may be potent candidates to activate DA neurons in the latVTA [11]. The findings suggest that GABA neurons in the NAc lat shell are activated via increased release of DA from latVTA DA neurons that were activated by the LDT–latVTA and LHA–latVTA pathways (Figure 4). Consequently, activation of GABA neurons in the NAc lat shell via activation of the mPFC–NAc, BLA–NAc, and latVTA–NAc pathways may inhibit GABA interneurons located in the latVTA (Figure 4(5)), which results in disinhibition of VTA DA neurons (Figure 4(6)) and, therefore, upregulation of DA release into the NAc lat shell (Figure 4(7)). All these events may be necessary to enhance the activation of the mesocorticolimbic system and may result in increased motivation to perform further VE. Taken together, greater activation and inhibition of the factors and the pathways presented in Figure 4(1) and Figure 4(7) may result in augmented EIH effects.

## 4. Attenuation of Fear Memories Contributes to EIH Effects

Chronic pain patients show anxiety, depression, and learning and memory deficits, which may be partially induced via atrophy and dysfunction of the HIP. Complex regional pain syndrome (CRPS) patients and chronic lower back pain patients showed significant decreases in HIP volume [21]. It has been demonstrated that the ability to extinguish contextual fear memories is significantly weakened in NPP model mice [20], and that neurogenesis in the hippocampal dentate gyrus is also inhibited in NPP model mice [21]. A projection pathway from the vHIP to the Amyg (vHIP–Amyg pathway) is suggested to play an important role in the reappearance of contextual fear conditioning and fear memory [44,45]. Accordingly, we examined whether VE can facilitate the attenuation of contextual fear memories in NPP model mice and observed that freezing times, indicating the strength of the fear memory, were significantly shortened in runner mice compared to non-runner mice. To investigate possible mechanisms that may explain this result, we injected Retrobeads red (RBR), a retrograde tracer, into the Amyg of sedentary mice. As a result, we detected many neurons co-expressing both FosB (a marker of activated neurons) and RBR (a retrogradely transported marker of neurons projecting to the Amyg from the vHIP) in the vHIP–CA region. Furthermore, we found that VE reduced NPP-induced activation of Glu neurons in the vHIP–CA1 region of NPP model mice, and that PV-positive GABA interneurons, which can inhibit pyramidal neurons located in the vHIP–CA1 region, were also activated in runner mice. These preliminary results suggest that inhibition of the vHIP–Amyg pathway via VE-induced activation of PV-positive GABA interneurons may facilitate the attenuation of contextual fear memories and could be a possible mechanism for the induction of EIH effects.

## 5. Conclusions

Brellenthin et al. reported that positive family environments predict attenuated pain sensitivity and greater EIH, whereas negative and chronic pain-present family environments predict worse pain and EIH outcomes. These findings indicate that psychosocial variables, such as the family environment and mood states, can affect both pain sensitivity and the ability to modulate pain through EIH [46]. Furthermore, pain catastrophizing has been found to attenuate EIH effects during exercise in healthy adults [47,48]. Less is known regarding the relationship between psychological factors and EIH effects in chronic pain patients. A few studies reported that psychosocial factors can influence the manifestation of EIH in normal adults, while they do not have an impact on EIH effects in chronic pain populations with musculoskeletal pain and fibromyalgia [49,50]. These findings suggest the possibility that factors other than psychosocial factors, such as the type, intensity, and duration of exercise, may play a role in the induction of EIH effects in chronic pain patients. Further research should be performed to determine certain relationships between EIH effects and psychosocial factors in chronic pain patients. The animal studies described here indicated that normalization of both the abnormal mesocorticolimbic system and the hyperactivated vHIP–Amyg pathway following VE in NPP model mice may be a reliable method for producing EIH effects. Therefore, suitable exercise in chronic pain conditions can activate the mesocorticolimbic system to trigger pain relief and a reduction in physical inactivity caused by fear and anxiety of movement-induced pain. These mechanisms may relieve chronic pain patients of “fear-avoidance belief” and facilitate EIH effects. The appropriate type, intensity, and duration of exercise required to enhance EIH effects have not yet been fully examined in animal and human studies. Furthermore, the first and foremost node that is activated by VE stimulation has not yet been specified in animals and humans. Therefore, further efforts to explore these issues may be critical to further understand the brain mechanisms underlying EIH effects.
